# Exploring subthreshold functional network alterations in women with phenylketonuria by higher criticism

**DOI:** 10.1186/s13104-026-07745-2

**Published:** 2026-02-24

**Authors:** Benedikt Sundermann, Reinhold Feldmann, Christian Mathys, Stefan Garde, Johanna M. H. Rau, Anke McLeod, Josef Weglage, Bettina Pfleiderer

**Affiliations:** 1 Institute of Radiology and Neuroradiology, Evangelisches Krankenhaus Oldenburg, Universitätsmedizin Oldenburg, Oldenburg, Germany; 2 Clinic of Radiology, Medical Faculty, University of Münster, Albert-Schweitzer-Campus 1, Building A1, 48149 Münster, Germany; 3 Research Center Neurosensory Science, Carl Von Ossietzky Universität Oldenburg, Oldenburg, Germany; 4 Department of General Pediatrics, University Hospital Münster, Münster, Germany; 5 Department of Psychiatry, Psychosomatic Medicine and Psychotherapy, University Medical Center Frankfurt, Frankfurt am Main, Germany; 6 Department of Nuclear Medicine, University of Leipzig, Medical Center Leipzig, Leipzig, Germany

**Keywords:** Phenylketonuria, Hyperphenylalaninemia, Cognition, Networks, Connectivity, Resting state fMRI

## Abstract

**Objective:**

Phenylketonuria (PKU) is an inherited disorder of amino acid metabolism. Despite early dietary treatment, cognitive functioning of patients has been reported as being inferior to healthy controls. Objective of this study was to assess functional connectivity (FC) alterations in PKU in cognition-related brain networks by resting-state functional magnetic resonance imaging. We followed a hierarchical analysis approach partially based on higher criticism (HC) statistics as previously applied in a larger sister-project in fetal alcohol syndrome.

**Results:**

After exclusions for excessive head movement, 11 female young adults with early-treated PKU (age: 27.2 ± 3.7 years) and 11 age-matched female healthy controls (age: 25.9 ± 3.8 years) were included in the analysis. We observed effects within attention networks and the default mode network, but not in fronto-parietal networks, at the HC-based intermediate analysis level. No between-network FC differences were found. In the most detailed analysis level, we could not identify single affected functional connections. Despite statistical power limitations in this small sample, these preliminary findings are in line with previously reported FC alterations in PKU and the cognitive profile in young adults with PKU, particularly the still uncertain notion that cognitive control deficits might become less pronounced when PKU patients reach adulthood.

**Supplementary Information:**

The online version contains supplementary material available at 10.1186/s13104-026-07745-2.

## Introduction

Classical phenylketonuria (PKU, OMIM 261600, https://www.omim.org/entry/261600) is an inherited metabolic disease affecting neurocognitive development [1–3]. If treated early by dietary intervention, most affected individuals reach normal or near-normal cognitive abilities [1, 2]. However, many PKU patients exhibit minor cognitive deficits, involving executive functions [4–7]. A recent meta-analysis of cognitive outcomes in adults with early treated PKU observed the most pronounced impairments in reasoning, visual-spatial attention, sustained attention, visuo-motor control, and flexibility [8].

Neuroimaging studies in early-treated PKU have mainly focused on structural alterations [9]. These include diffuse T2w white matter hyperintensities (WMH), predominantly in parieto-occipital regions, but also locally reduced cortical thickness and alterations to white matter tracts [9]. An anterior–posterior gradient has been observed in such morphometric studies [10, 11], suggesting a potential association with the spatial distribution (posterior dominance) of WMH. Functional neuroimaging studies in PKU, including functional magnetic resonance imaging (fMRI) have focused on task-based protocols examining higher cognitive functions [12–16]. Knowledge about cerebral alterations from a functional network perspective is, however, still very limited. Christ et al. observed decreased functional connectivity in the default mode network (DMN) by resting state fMRI (rs-fMRI) in a small and very heterogeneous PKU sample [17]. Potential DMN alterations could also be indirectly inferred from interpretations of altered activity patterns in task-based fMRI [12]. Further evidence of altered functional connectivity (FC) or network dysfunction in PKU results from a study in task-based FC of the prefrontal cortices [14]. Another rs-fMRI study is ongoing [18].

Reliably assessing and interpreting functional neuroimaging data in diseases like PKU is challenging. Generally, sample sizes in neuroimaging studies in affected individuals are limited by the rarity of this inherited metabolic disorder affecting neurocognitive development [1, 9], similar to other neurodevelopmental disorders [19]. This scarcity of data points limits the detectability of disease-related alterations in conventional fMRI analyses, which commonly employ mass-univariate statistical testing [20]. Statistical power limitations in such studies also impair the reliability of published findings [21–24]. In the aforementioned research scenario, few strongly localized statistically significant findings might frequently not well represent more distributed underlying effects at the level of functional networks [20, 21]. Therefore, complementary methods taking wider patterns of activation or connectivity estimates (both above and below conventional statistical thresholds) into account [20] might be better suited to elucidate robust network alterations in the aforementioned patient groups. Among several approaches to explore subthreshold effects in fMRI, analyses using higher criticism (HC) statistics have been suggested [20]. HC is a method to identify rare and weak effects in high-dimensional data [25–27]. It follows the rationale of p-value histogram interpretation to detect an excess of low p-values in a range of primary tests in order to reject a global null hypothesis [28].

We recently reported the application of multi-scale FC modelling based on HC statistics in fetal alcohol syndrome (FAS): This approach identified subnetworks containing FC group differences while single altered connections could not be reliably identified using conventional analysis methods [29]. Here, we applied this analysis approach [29] to a substantially smaller sample of young women with PKU. The small sample size, resulting from necessary exclusions for excessive head motion in the MRI scanner [30] and the rarity of PKU [1], would not be regarded as sufficient for conventional rs-fMRI analyses. We therefore consider this to be exploratory work and thus intentionally decided to publish it in this specific format, rather than as a classical research article in a disease-specific journal.

The primary objective of this study was to examine FC in brain networks related to cognition in young adult women with early-treated PKU. The following hypotheses were tested, hereby conceptually following the approach previously reported [29]: (1) FC in the connectome of all brain regions representing brain networks related to cognition is altered in PKU compared with a control group of healthy participants. (2) FC within individual cognition-related brain networks is altered in PKU compared with healthy participants (network-wise global hypotheses and individual connections). (3) FC between cognition-related brain networks is altered in PKU compared with healthy participants (global hypothesis and individual connections).

## Materials and methods

This study was part of a larger functional neuroimaging project in PKU and FAS with overlapping samples [12, 29, 31]. PKU-related aspects of this study follow a concomitant task-based fMRI study [12], and fMRI methodology follows the related study in FAS [29] within this project. Related project details are thus only briefly summarized here.

### Subjects

Primary inclusion and exclusion were identical with the task-based fMRI study in PKU and have been published there [12]. The exclusion criterion of participation in an earlier task-based fMRI study on inhibitory control in males with PKU [13] eventually limited the sample to women, since no sufficiently large group of male subjects with PKU could be recruited. 17 patients attended the MRI examination. Complete MRI data could be acquired in 15 patients. Further participants were excluded from the analyses because of potentially biasing lesions (identified by structural imaging), psychoactive medication, and impaired MRI data quality before HC-based group analyses (criteria identical with the accompanying rs-fMRI study in FAS [29]): Data from further 4 PKU patients were excluded because of excessive head motion (2 based on quantitative criteria, 2 based on visual quality control criteria/carpet plots, see below). The remaining 11 PKU subjects were included in the final analyses. All PKU patients received early dietary treatment according to the then-current recommendations of the German working group on pediatric metabolic disorders [32].

An identical number (n = 11) of female healthy controls were drawn from the control group in the PKU/FAS neuroimaging research project [12, 29, 31] by age-based matching. This was necessary since the control group was on average older than the PKU group. Control subjects fulfilled the same inclusion and exclusion criteria as the PKU group, except the primary diagnosis.

As in the related FAS study [29] questionnaires included the Edinburgh Handedness Inventory (EHI) [33] and a trail-making task (TMT) of processing speed [34].

Demographical data and test results are presented in Table 1.

**Table 1:** Demographical and clinical characteristics of female PKU patients (n = 11) and controls (n = 11)

	Group	Mean ± SD	Median	Range	p^a^
Age (years)	PKU	27.2 ± 3.7	27	20 – 31	0.478
	CON	25.9 ± 3.8	26	20 – 32	
EHI Handedness index	PKU	87.7 ± 7.3	91.7	72.7 – 91.7	0.243
	CON	85.5 ± 8.4	83.3	75.0 – 100	
TMT reaction time (sec)	PKU	77.5 ± 48.5	64.0	43.0 – 217.0	0.088
	CON	52.5 ± 10.7	47.8	35.5 – 69.5	
Time to treatment onset (years)^b^	PKU	0.077 ± 0.066	0.08	0.02 – 0.23	NA
	CON	NA	NA	NA	
Childhood [Phe]_serum_^c^ (mg/dl)	PKU	6.7 ± 1.8	6.8	3.9 – 9.7	NA
	CON	NA	NA	NA	
Latest [Phe]_serum_^d^ (mg/dl)	PKU	14.5 ± 7.26	14.2	6.1 – 27.7	NA
	CON	NA	NA	NA	
WMH score	PKU	5.8 ± 1.7	7	2—7	NA
	CON	NA	NA	NA	

^a^Mann-Whitney-U-test

^b^available in n = 9

^c^average of available values during the first six years of life, available in n = 10

^d^median (range) in years before the fMRI measurement: 0.43 (0.0 – 3.0). PKU: phenylketonuria, CON: controls, SD: standard deviation, NA: not applicable, EHI: Edinburgh Handedness Inventory, TMT: trail-making task, Phe: phenylalanine; WMH: white matter hyperintensities

### Acquisition and analysis of MRI data

Data were acquired with a 3 Tesla scanner including 9:45 min of rs-fMRI data (gradient-echo echo planar imaging, 234 functional volumes after 5 non-recorded dummy scans; repetition time: 2500 ms, echo time: 35 ms, 36 axial slices, spatial resolution: 3.6 × 3.6 × 3.6 mm, field of view: 230 mm), T1-weighted 3D data as reported previously [29], and T2-weighted 2D fluid-attenuated inversion recovery (FLAIR) [12]. WMH in PKU were visually scored on FLAIR images by a radiologist (BS) [35].

fMRI data preprocessing was identical with the FAS study [29] and was mainly based on fMRIPrep [36] (RRID:SCR_016216) with subsequent denoising [37]. MRI data quality control comprised visual inspection of structural data and preprocessing reports as well as exclusion of subjects with excessive head motion based on motion parameter estimates (mean frame-wise displacement, FD > 0.3 mm or maximum FD > 5 mm or more than 20% outlier data points) and visual quality indicators (motion-related sudden transitions in the fmriprep carpet plots) identical to the FAS study [29]. There was no significant group difference of head movement (see Additional file 1: Table S1).

FC analyses were based on 400 atlas regions [38] matched to 17 subnetworks [39]. FC between regions in 10 cognition-related subnetworks [29] was calculated [40]. Further analyses also followed our previously published approach [29]: As a basis for subsequent modelling, multiple linear regression models were calculated (one model per pair of atlas regions), comparing z-transformed correlation coefficients (dependent variable) among regions between PKU subjects and healthy participants. Group (PKU vs. control, categorical), age (normalized to center: 0, and standard deviation: 1), mean framewise-displacement (FD) representing head motion, and a constant term were defined as independent variables. Main effects of group (PKU/control, two-sided) were the basis of the subsequent analyses at different spatial scales, analogously to the FAS study [29]: This subsequent hierarchical analysis comprised the following steps: first, determining whether there is any at least rare or weak alteration of FC in PKU in the full cognitive connectome globally (using HC) and subsequently aiming to resolve these findings: at the level of either FC within each network or between-network (concatenated regions per network) connectivity (using HC), and finally at the most detailed level of individual connections (conventional mass-univariate analysis with false-discovery rate (FDR) adjustment [41] for multiple comparisons). Please refer to the previous study for further model and software implementation details [29].

## Results

**Fig. 1:**
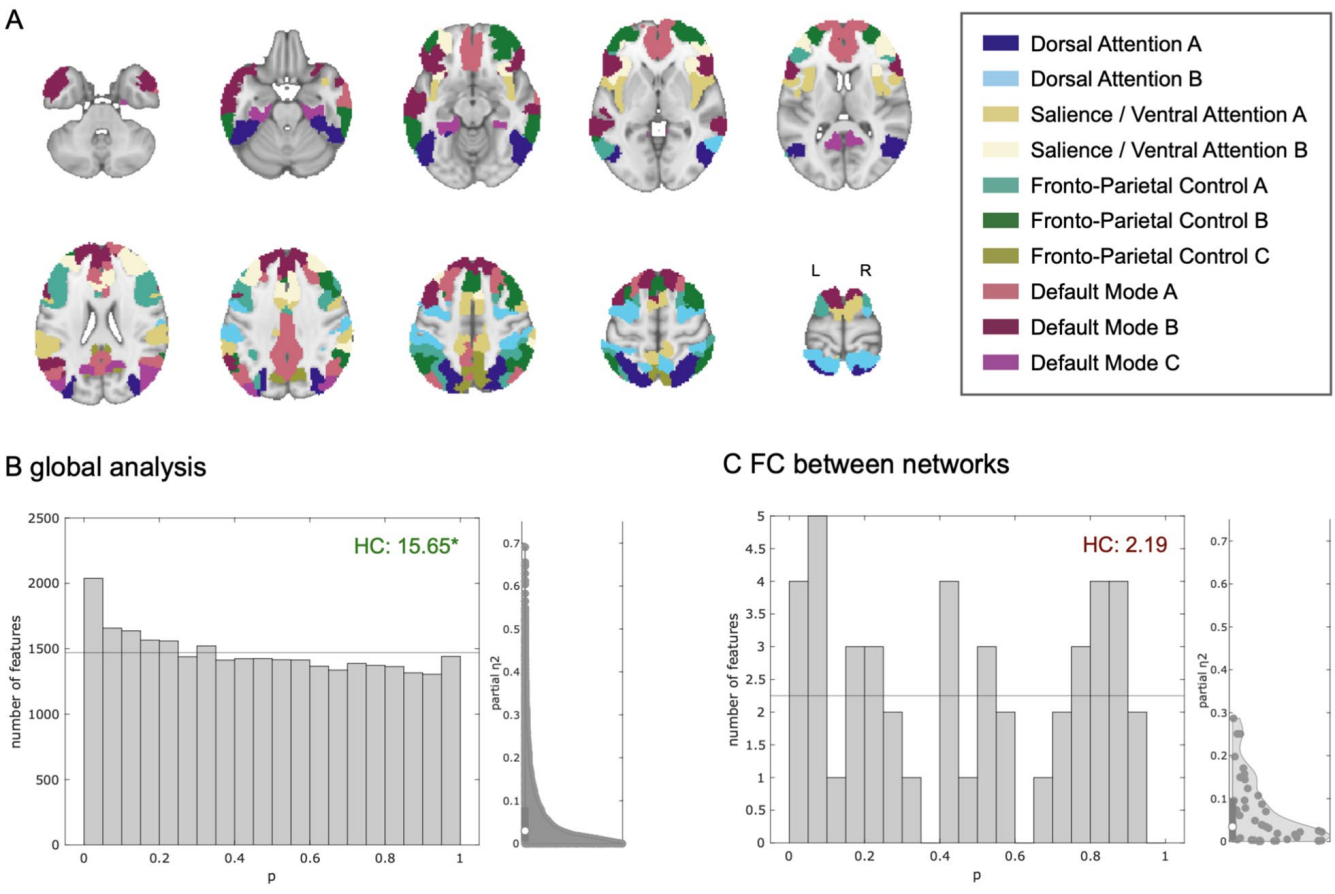
Global analysis and between-network functional connectivity (FC) differences of cognition-related brain networks. **A** Overview of the ten cognition-related sub-networks based on the cortical atlas used in this analysis (previously published in [29] under a creative commons license: http://creativecommons.org/licenses/by/4.0/). **B** P-value histogram and half violin plot of standardized effect size estimates of multiple individual linear models (main effect of group) comparing FC among all 243 single regions between PKU patients and healthy control subjects. Under the null-hypothesis of equal FC in both groups, equal numbers of p-values (horizontal line) are expected in each bin. The histogram shows an excess of low p-values. The existence of at least rare and/or weak effects visualized in the histogram is confirmed by a quantitative test of the joint hypothesis based on higher criticism statistics. This means that regarding a significant number of functional connections, PKU patients differ from healthy controls. **C** The null-hypothesis could not be rejected in the between-network connectivity analysis. HC: higher criticism test statistic (green: global null hypothesis rejected; red: global null hypothesis not rejected)

The global hypothesis test (HC) in the full cognitive connectome (i.e. connections between individual brain regions) revealed at least rare and weak group differences between this sample of PKU patients and the healthy control group (Fig. 1B). The global null hypothesis for between-network connectivity could not be rejected (Fig. 1C).

**Fig. 2:**
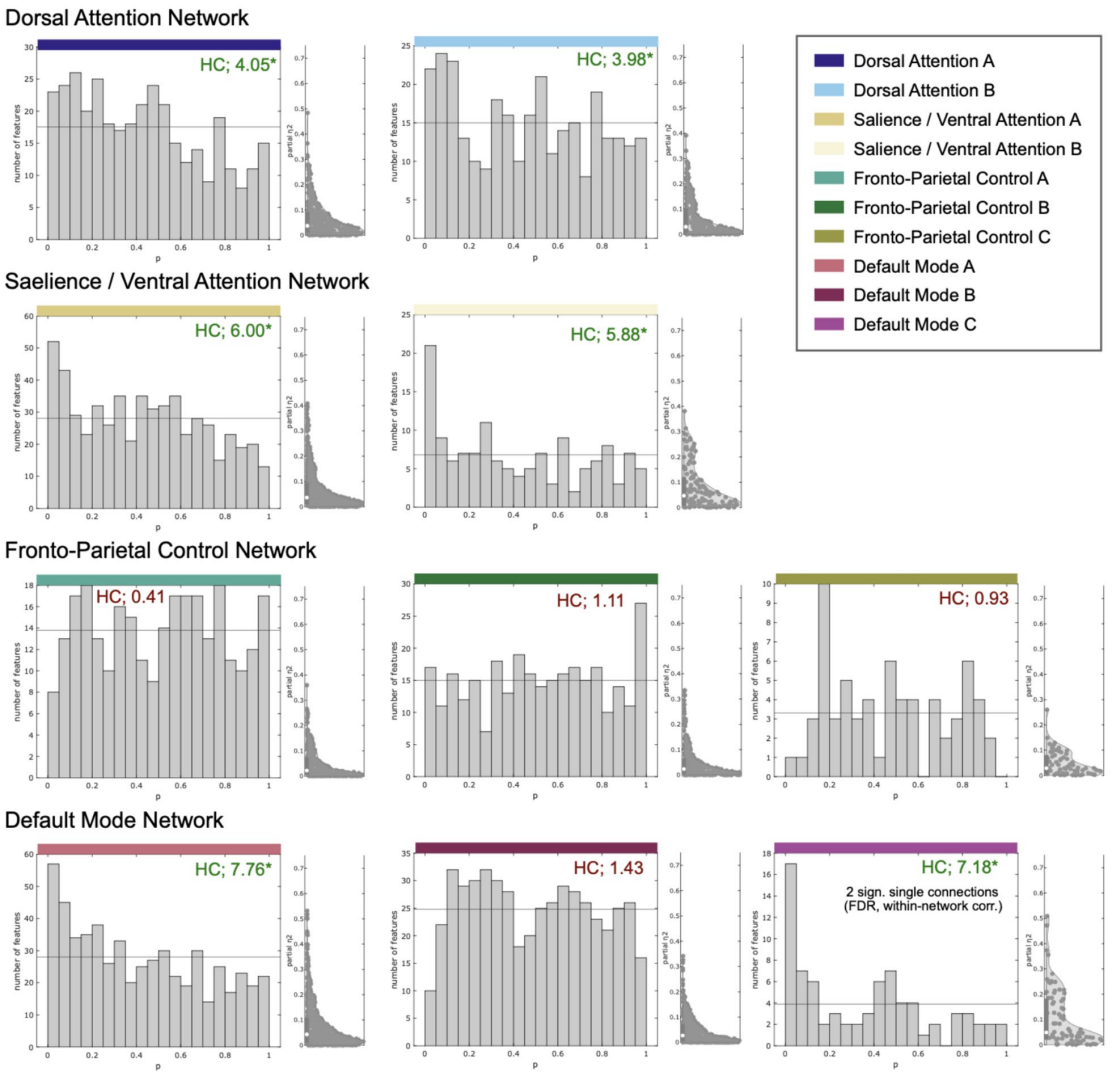
Within-network functional connectivity differences in cognition-related brain networks. Six (out of ten) sub-networks exhibited altered functional connectivity in PKU patients compared with controls (p-value histograms and half violin plots of standardized effect size estimates): All four attention-related sub-networks and two sub-networks of the default mode network exhibited an excess of low p-values of multiple linear models (main effect of group) compared with the expected number under the global null hypotheses (horizontal line). This means that the PKU patients differed from healthy controls regarding at least rare and/or weak effects within these networks. However, subsequent tests for individual connections were not statistically significant with global multiple comparison correction. HC: higher criticism test statistic (*green: global null hypothesis rejected; red: global null hypothesis not rejected)

In the network-specific within network-analyses (HC-based) group differences in FC were observed within 6 out of 10 cognition-related networks (Fig. 2). Based on the HC statistic, differences were most pronounced in 2 out of 3 subcomponents of the DMN (A and C; no effects observed in subnetwork B). Beyond, we found effects within all 4 attention- and salience-related subnetworks. In contrast, no effects were observed within the three subcomponents of the fronto-parietal control network.

Two single connections within the DMN C sub-network exhibited significant group differences with FDR-adjustment within this network only. These findings did, however, not survive FDR-correction across all connections.

## Discussion

Using an HC-based exploratory rs-fMRI analysis method [29], we were able to observe alterations of FC in young adult females with early-treated PKU when compared to healthy individuals. Despite the small sample size, these preliminary results follow an interpretable pattern which show group differences attributable to attention-related functional networks and the DMN.

The involvement of the DMN is in line with limited previous fMRI results [12, 17]. The observed involvement of only the 2 DMN subnetworks (A and C) with substantial share of posterior brain regions corresponds to the typical distribution of WMH [9] as well as previous morphometric findings [10, 11, 42]. By visual comparison of atlas regions, the DMN A subnetwork substantially overlaps with the previously proposed DMN midline core, while the DMN C subnetwork mainly overlaps with the DMN medial temporal lobe subsystem [43].

To date, there is no previous fMRI evidence of altered attentional systems in PKU. However, this observation is in line with the neuropsychological profile (i.e. involving impairments in visual-spatial attention, and sustained attention,) reported in adults with early treated PKU [7, 8]. In this study, group differences appear pronounced in the salience/ventral attention networks compared with the dorsal attention network.

The negative finding in the fronto-parietal control network appears surprising in the context of the classical assumption of prefrontal specificity of deficits in children with PKU [44, 45]. Executive control deficits have also been reported in adults with PKU as part of a wider spectrum of neurocognitive deficits [4–6, 8]. The absence of findings in the fronto-parietal networks in this study might, however, correspond with a potential regression of certain PKU-related deficits during aging [7, 46–48]. It is also in line with previous task-based fMRI studies with negative [13] or only weak findings [12, 15] in fronto-parietal control networks in adults.

The comparatively straightforward interpretability of findings and correspondence with previous knowledge might indicate a potential benefit of a network-wise HC analysis in rare diseases, to which the previously discussed limitations (such as small sample sizes) apply.

In conclusion, this sample exhibited FC alterations in networks which are in line with limited previous neuroimaging findings and the clinical neuropsychological profile in adult PKU patients. However, given the small sample size, exploratory character, and lack of significant findings in the final most detailed analysis level, these observations should be interpreted with care and confirmation in future studies in larger PKU samples should be attempted.

## Limitations

Application of HC to FC-analyses in this or similar ways [29, 49, 50] is a relatively novel approach with limited knowledge compared with conventional mass-univariate fMRI analyses. However, there is conceptual overlap with the more established method of network contingency analysis [51]. Methodological limitations have been discussed in detail in our related FAS article [29].

The generalizability of our observations is limited by the small sample size, restriction to women in a relatively narrow age range and TMT performance differences between PKU and controls (potentially related to IQ differences [34, 52]), as well as by a limited clinical, neuropsychological, and social characterization of the sample. Additionally, more detailed information about dietary control throughout life, and in particular in adulthood, would have been desirable [53, 54]. Beyond, sample size and statistical power restrict the ability to identify which exact connections were altered and how exactly they were affected (including potential associations between recent Phe-levels representing dietary control and FC alterations). This should be considered in future neuroimaging research in larger PKU samples. Our subnetwork-level results might, however, be informative for planning and interpreting future larger scale neuroimaging studies in PKU, e.g. by narrowing down hypotheses to the affected subnetworks observed here.

## Supplementary Information

Additional file 1.

## Data Availability

Due to data protection regulations and to safeguard participant confidentiality, we cannot share primary data (no participant consent for individual-level data sharing). Further aggregate data on an intermediate level can be shared on reasonable request to the authors. This work is based on a combination of widely used commercially available software as well as publicly available software and statistical algorithms, either referenced in the methods section or the more detailed description in our previous article [29]. Further in-house code was used for individual aspects of data handling only. This could be shared on reasonable request to the authors.

## Abbreviations

DMN: Default mode network
FAS: Fetal alcohol syndrome
FC: Functional connectivity
FD: Frame-wise displacement
FLAIR: Fluid-attenuated inversion recovery
fMRI: Functional magnetic resonance imaging
HC: Higher criticism
MRI: Magnetic resonance imaging
OMIM: Online Mendelian Inheritance in Man
PKU: Phenylketonuria
rs-fMRI: Resting state functional magnetic resonance imaging
TMT: Trail-making task
WMH: White matter hyperintensities
