# Supplementary material for: Exploring subthreshold functional network alterations in women with phenylketonuria by higher criticism
Source: BMC Res Notes. 2026 Feb 24;19:153. doi: 10.1186/s13104-026-07745-2 (PMC13041494; doi:10.1186/s13104-026-07745-2)
Supplement: Supplementary file 1 — Additional file 1. [file 13104_2026_7745_MOESM1_ESM.pdf]

Supplementary material

Supplementary Table 1 – Group comparison of head motion estimates

Data derived from preprocessing (final sample after exclusion of subjects with excessive head motion).

	PKU[a]	CON[a]	p[b]
Mean FD (mm)	0.167 (0.10 – 0.28)	0.150 (0.12 – 0.25)	0.898
Maximum FD (mm)	0.865 (0.25 – 1.75)	0.946 (0.55 – 1.65)	0.300
Percentage of motion spikes	1.282 (0.00 – 8.97)	1.709 (0.43 – 6.84)	0.519

[a] median (range) [b]Mann-Whitney-U-test result. PKU: phenylketonuria, CON: controls, FD: framewise displacement
